# Protection against mycobacterial infection: A case-control study of mycobacterial immune responses in pairs of Gambian children with discordant infection status despite matched TB exposure

**DOI:** 10.1016/j.ebiom.2020.102891

**Published:** 2020-07-13

**Authors:** Robindra Basu Roy, Basil Sambou, Muhamed Sissoko, Beth Holder, Marie P Gomez, Uzochukwu Egere, Abdou K Sillah, Artemis Koukounari, Beate Kampmann

**Affiliations:** a Department of Academic Paediatrics, Section of Paediatric Infectious Disease, Imperial College London, St. Mary's Hospital, Praed Street, London W2 1NY, United Kingdom; b Vaccines and Immunity Theme, MRC Unit The Gambia at the London School of Hygiene and Tropical Medicine, Atlantic Road, Fajara, The Gambia; c Clinical Research Department, Faculty of Infectious and Tropical Diseases, London School of Hygiene and Tropical Medicine, Keppel Street, London WC1E 7HT, United Kingdom; d Institute of Reproductive and Developmental Biology, Department of Metabolism, Digestion & Reproduction, Imperial College London, Du Cane Road, W12 0HS, United Kingdom; e Department of International Public Health, Liverpool School of Tropical Medicine, Pembroke Place L3 5QA, United Kingdom; f Department of Infectious Disease Epidemiology, Faculty of Epidemiology and Population Health, London School of Hygiene and Tropical Medicine, United Kingdom; g The Vaccine Centre, London School of Hygiene and Tropical Medicine, Keppel Street, London WC1E 7HT, United Kingdom

**Keywords:** Paediatric, Tuberculosis, Latent tuberculosis infection, Correlates of protection, Mycobacterial growth inhibition assay

## Abstract

**Background:**

Children are particularly susceptible to tuberculosis. However, most children exposed to *Mycobacterium tuberculosis* are able to control the pathogen without evidence of infection. Correlates of human protective immunity against tuberculosis infection are lacking, and their identification would aid vaccine design.

**Methods:**

We recruited pairs of asymptomatic children with discordant tuberculin skin test status but the same sleeping proximity to the same adult with sputum smear-positive tuberculosis in a matched case-control study in The Gambia. Participants were classified as either Highly TB-Exposed Uninfected or Highly TB-Exposed Infected children. Serial luminescence measurements using an *in vitro* functional auto-luminescent Bacillus Calmette–Guérin (BCG) whole blood assay quantified the dynamics of host control of mycobacterial growth. Assay supernatants were analysed with a multiplex cytokine assay to measure associated inflammatory responses.

**Findings:**

29 pairs of matched Highly TB-Exposed Uninfected and Highly TB-Exposed Infected children aged 5 to 15 years old were enroled. Samples from Highly TB-Exposed Uninfected children had higher levels of mycobacterial luminescence at 96 hours than Highly TB-Exposed Infected children. Highly TB-Exposed Uninfected children also produced less BCG-specific interferon-γ than Highly TB-Exposed Infected children at 24 hours and at 96 hours.

**Interpretation:**

Highly TB-Exposed Uninfected children showed less control of mycobacterial growth compared to Highly TB-Exposed Infected children in a functional assay, whilst cytokine responses mirrored infection status.

**Funding:**

Clinical Research Training Fellowship funded under UK Medical Research Council/Department for International Development Concordat agreement and part of EDCTP2 programme supported by European Union (MR/K023446/1). Also MRC Program Grants (MR/K007602/1, MR/K011944/1, MC_UP_A900/1122).

**Research in context**

**Evidence before this study**

Over one million children develop tuberculosis each year, often through household exposure to an adult with pulmonary tuberculosis. However, most children with such exposure develop neither tuberculosis disease, nor latent tuberculosis infection (LTBI) and these individuals are important to study to understand protective immunity.

Studies using functional whole blood mycobacterial growth assays have shown superior growth control by adults with LTBI compared to healthy controls, whilst another study including children found no difference. Production of the cytokine interferon-γ (IFNγ) is the basis of blood tests for LTBI, whilst Tumour-Necrosis Factor-α (TNFα) production has also been linked to responses in the tuberculin skin test for LTBI. However, differences in TNFα responses to mycobacterial stimulation have not been consistently found in household TB contact studies.

Search strategy: PubMed was searched using the terms “immun* AND ("latent tuberculosis" OR "tuberculosis infection")” with a filter of “Child: birth-18 years” and no date or language limitations on 10/6/20.

**Added value of this study**

To our knowledge, this is the first study of children with high exposure to TB but who have not developed disease or LTBI that has used an exposure-matched case-control design. We recruited pairs of asymptomatic children with discordant tuberculosis infection status but the same sleeping proximity to the same adult with sputum smear-positive tuberculosis in The Gambia. Using a whole blood autoluminescent mycobacterial growth assay we found that High TB-Exposure Uninfected children had less control of mycobacterial growth at 96 h than matched High TB-Exposure Infected children. Multiplex cytokine analysis of supernatants from this assay showed interferon-γ responses reflected LTBI status but no significant differences in TNFα and other measured cytokines.

**Implications of all the available evidence**

Children with effective immunity against tuberculosis infection are an important study population to try to identify correlates of protection that can guide vaccine design and evaluation. *In vitro* experiments using clinical samples that focus on gene expression, inflammatory and innate immune responses, and antibody effector function may be helpful in future studies.

Alt-text: Unlabelled box

## 1 Introduction

The WHO have set the ambitious goal of ending TB by 2035 and achieving zero childhood TB deaths [1,2]. Key to this is the development of effective vaccines, which requires identification of correlates of protective immunity [1,[3], [4], [5]]. Children represent a particularly important and interesting population, as although young children are susceptible to disease progression and have high morbidity and mortality, early school-age children appear to be relatively protected against development of tuberculosis, and some children show no signs of disease or asymptomatic infection despite defined exposure to infectious cases of pulmonary tuberculosis [5,6]. Latent tuberculosis infection (LTBI) is a clinically relevant phenotype because the risk of incident tuberculosis is significantly higher in children of all ages with a positive baseline tuberculin skin test (TST) or Interferon Gamma Release Assay (IGRA) compared to those with negative baseline tests [7,8]. Building on paediatric observational data, there is now clinical trial evidence that Bacillus Calmette–Guérin (BCG) vaccine protects against TB infection [9], [10], [11]. This highlights that it is a) possible to induce a protective immune response and b) the importance of studying those who have neither tuberculosis disease nor infection despite exposure to *M tuberculosis* (MTB).

Nevertheless, the biological basis of both natural and vaccine-inducible human protection against TB infection remains poorly understood [12]. Genetic loci associated with TST responses overlap with a region associated with Tumour Necrosis Factor-α (TNFα) production [13], [14], [15], [16], [17]. Enhanced antibody avidity and distinct MTB-specific IgG Fc profiles as well as a role for NK cells, and changes in innate immune cell subpopulations over time have all been implicated [18], [19], [20], [21], [22], [23]. Identification of such correlates of protective human immunity against infection are critical to design and evaluation of novel vaccines for prevention of infection and to shorten the duration of costly clinical trials [24].

One potential correlate of protection is mycobacterial growth inhibition by either whole blood or peripheral blood mononuclear cells [25], [26], [27], [28]. The BCG-lux assay, a functional mycobacterial growth inhibition assay, employs reporter-gene tagged mycobacteria [29,30]. In this assay, a growth ratio is calculated based on luminescence of recombinant mycobacteria following lysis of *in vitro* infected whole blood samples at baseline and 96 hours. An alternative Mycobacterial Growth Inhibition Assay (MGIA) uses readouts of time to positivity in automated BD BacTec culture systems following inoculation of a blood sample with mycobacteria [25,26]. Both the BCG-lux assay and the BacTec-based MGIA provide a single numerical value as the result and do not capture the dynamics of early host-mycobacterial interactions.

Household studies of contacts of a TB index case are a useful epidemiological platform through which to investigate the spectrum of immune responses to MTB exposure [31]. Such studies generally compare groups of participants with a particular phenotype from the study population. Interpretation of results is complicated by variation in infectiousness of the source case, duration and intensity of the interaction, infectivity of the organism, and the child's immune status [5,[32], [33], [34], [35]]. We therefore instead conducted a matched case-control study nested within an established household contact platform in The Gambia [31,34,[36], [37], [38]]. We recruited pairs of asymptomatic children with discordant TST despite the same sleeping proximity to the same smear-positive adult with recently diagnosed pulmonary tuberculosis. This study design therefore minimises confounding as within each pair the children will have been exposed to the same strain of MTB from the same adult with the same index case characteristics. We recruited participants older than five years old, to focus on the age range where children are relatively protected against TB disease. This also prevented confounding through isoniazid preventive therapy provision for all household contacts under five in keeping with WHO recommendations [5,6,38].

We also developed a novel autoluminescent recombinant BCG whole blood mycobacterial growth inhibition assay with serial, non-destructive measurement of luminescence on the same sample, thereby enabling study of the dynamics of the host-mycobacterial interaction using minimal blood volumes [39]. This method also enables biochemical analysis of supernatants from the BCG-infected whole blood samples to evaluate the profile of pro- and anti-inflammatory cytokine responses implicated in host control of mycobacteria such as interferon γ (IFN-γ), TNFα, Interleukin-1α and β (IL-1α, and IL-1β), and IL-10 [40]. We hypothesized that Highly TB-Exposed Uninfected children would be better able to restrict mycobacterial growth at early time points in the *in vitro* assay than their matched Highly TB-Exposed Infected counterparts. We also hypothesised that Highly TB-Exposed Uninfected children would have a pro-inflammatory profile at early timepoints in the assay (with high levels of TNFα, IL-1α, and IL-1β, and low levels of IL-10) associated with an effective innate immune response, whilst the adaptive response of Highly TB-Exposed Infected children would lead to elevated levels of IFN-γ at later timepoints.

## 2 Materials and methods

*Recruitment:* All household compounds that were originally enroled in the childhood TB household contact platform at MRC Unit The Gambia between 1st January 2015 and 31st December 2015 were evaluated for inclusion in this study [34,[36], [37], [38]]. Briefly, adult index cases (>15 years old) with newly identified smear-positive pulmonary tuberculosis were approached for consent to visit their household compounds. A compound was defined as a cluster of homes or buildings often owned by members of the same family, with typically 7–8 child contacts per index case [34]. Families within this compound were then consented for their children <15 years old to be screened for symptoms of tuberculosis and a TST. Those with a TST ≥10 mm or symptoms were referred to clinic for evaluation. All participants were screened for symptoms of tuberculosis 3-monthly for one year. Neonatal BCG immunisation coverage in The Gambia is estimated at 98% [41].

*Sample size calculation:* This was based upon unpublished pilot data from children with tuberculosis exposure using the BCG-lux assay (Supplementary Fig. 1). Assuming a paired test to allow for matching, data were bootstrapped with age matching. Taking 10,000 bootstrapped samples of size 30 in each group gave a power of 80% for demonstrating a significant difference for a two-sided non-parametric Wilcoxon signed-rank test at the 5% level.

*Case definitions:* Case definitions and inclusion and exclusion criteria that were used to identify Highly TB-Exposed Infected and Highly TB-Exposed Uninfected children for this study are summarised in Supplementary Tables 1 and 2 [34]. Matched pairs of children had the same sleeping proximity in the same building to the same adult with smear-positive pulmonary tuberculosis. Children aged less than 5 years old were excluded as they were prescribed isoniazid preventive therapy in line with WHO recommendations [38]. Participants in this study were not treated for LTBI in keeping with WHO guidelines at the time in view of their age and the absence of HIV infection [42]. All participants were asymptomatic at the time of initial household symptom screening and were recruited to this study at least 3 months after the initial screening for a repeat TST to confirm that the children identified as Highly TB-Exposed Uninfected had clear evidence of having remained uninfected (TST ≤5 mm). Highly TB-Exposed Infected children had an initial TST ≥10 mm in keeping with WHO thresholds for a positive TST, a negative HIV test, a normal chest radiograph, and a normal physical examination by a paediatrician [42]. An in-house IGRA was taken from Highly TB-Exposed Infected children at this initial clinic visit. Where more than one child in the compound met the criteria for being Highly TB-Exposed Uninfected or Highly TB-Exposed Infected, a single child from those eligible was selected at random. Demographic and exposure data were collected by the study fieldworker. The consent process included permission to access the HIV status of the tuberculosis index case that had been tested as part of routine clinical care in this low HIV prevalence setting [34].

*Sample procedures:* Venous blood from matched Highly TB-Exposed Uninfected and Highly TB-Exposed Infected children was collected at the same visit. Samples were collected using lithium heparin tubes for the whole blood assay and EDTA tubes for full blood count (both BD, Oxford, UK). Full blood counts were analysed by the Clinical Services Diagnostic Haematology Laboratory at MRC Unit The Gambia at LSHTM using the Cell Dyn 3700 Haematology Analyser (Abbott Diagnostics, Illinois). If this was not available, full blood count samples were run using Medonics M-series (Boule Medical AB, Spånga, Sweden) in the Immunology Research Laboratory. Samples from both members of a pair were always analysed with the same method. Monocyte: lymphocyte ratios were also calculated [43]. In-house interferon gamma release assays on Highly TB-Exposed Infected children were conducted as has previously been described [36,44,45].

*Autoluminescent BCG growth monitoring in whole blood:* The method has been fully reported [39]. In brief, BCG Danish was transformed to express the Luciferase Full Operon of *Photorhabdus luminescens* (RRID:Addgene_49999 with RRID: Addgene_50000) and green fluorescent protein (RRID:Addgene_30173) with a ratio of 0.05 Relative Light Units(RLU)/ml/s: 1 Colony Forming Unit (CFU). Aliquots were grown to logarithmic growth phase in liquid culture with antibiotic selection markers (kanamycin 20 μg/ml [Glyco/Life Technologies, Carlsbad, CA, USA] and hygromycin 50 μg/ml [Sigma-Aldrich, Gillingham, UK]) and diluted to 3.3 × 10^5^ RLU/ml/s immediately prior to venepuncture. Samples from both members of a pair were always analysed in the same experiment. Heparinised whole blood was diluted 1:1 with RPMI 1640 culture medium (Sigma-Aldrich, Gillingham, UK) containing 2.5% 1 M HEPES buffer and 1% l-glutamine (both Sigma-Aldrich). BCG-GFP-LuxFO was added at a ratio of 1 part BCG-GFP-LuxFO: 9 parts whole blood in culture medium (equivalent to 1.3 × 10^5^ CFU per 100 μL undiluted whole blood). For the control samples, bacterial medium alone was added in the same ratio of 1:9. 500 μl was aliquoted in triplicates for each experimental condition at each timepoint into sterile lidded 75 × 12 mm polystyrene tubes (Corning B.V Life Sciences, Amsterdam, The Netherlands) and transferred to a rocking incubator at 37 °C. Luminescence readings of each tube were measured immediately after removal from the incubator using a Sirius Tube Luminometer (Berthold Detection Systems GmbH, Pforzheim, Germany) 1, 4, 24, 48, 72 and 96 hours after the bacteria were added, and corrected for background luminescence. The baseline luminescence reading was taken one hour after inoculation of samples to ensure equilibration to a temperature of 37 °C in the incubator [39]. In addition, at baseline (0 h), 24 hours, and 96 hours, further sets of triplicate samples were centrifuged at 2000 x g for ten minutes and supernatants stored at −70 °C and shipped on dry ice to Imperial College London for cytokine analysis. Experimenters were not blinded to group assignment for the experimental work.

*Cytokine analysis:* Triplicate supernatant aliquots were thawed, pooled, and sterile filtered by spinning for 10 min at 5000 RPM with 0.22 µm centrifuge Spin-X tube filters (Corning B.V Life Sciences, Amsterdam, The Netherlands), transferred to 96 well plates and stored at −70 °C prior to analysis. A standardised custom commercial multiplex magnetic bead-based immunoassay, Bio-Plex Pro (Bio-Rad, Hercules, CA, USA), was used to quantify levels of IL-1α and IL-1β, IL-10, IFN-γ, TNF-α following the manufacturer's instructions. All samples from matched Highly TB-Exposed Infected / Highly TB-Exposed Uninfected pairs were run on the same plate and read using a Luminex 200 plate reader (LuminexCorp, Austin, Tx, USA). Quality control samples made up of pooled supernatants from non-study assays were run in duplicate on each plate and a normalisation factor applied to ensure comparability between plates. Samples falling below the quantification level of the assay were allocated values ¼ of the lower limit of detection.

*Statistical analysis:* We used normal quantile plots and Shapiro-Wilk tests to assess data distribution. To conduct unadjusted bivariate tests for association, the matched paired *t*-test was used for normally distributed data, Wilcoxon's matched pairs signed rank test for non-normally distributed data, and McNemar's χ^2^ test for binary variables. To examine multivariable associations of luminescence data multi-level linear regression (or mixed effects linear modelling) was used [46]. Luminescence data was log transformed due to the skewness of the data. The matched pairs and the individual participants were considered as random effects, whilst Highly TB-Exposed Uninfected / Highly TB-Exposed Infected status, age (as a categorical variable in tertiles), being a sibling of the adult index case and experimental time point were considered as fixed effects, the combination of all of which were used to predict log luminescence (dependant variable). Estimated marginal means and 95% confidence intervals for luminescence by group and time were predicted from the model treating all fixed effects including age and whether the child was a sibling of the adult index case as balanced. Statistical interpretation of differences between groups at each time point was carried out through pairwise comparisons [46]. STATA Statistical Software: Release 12.1. (StataCorp LP, College Station, TX) and Prism 7 for MacOS X (Graphpad Software Inc) were used.

*Ethical approvals:* The study was approved by The Gambia Government/MRC Joint Ethics Committee (SCC1405 and SCC1273) and the Imperial College Healthcare Tissue Bank (R13071).

## 3 Results

### 3.1 Participant characteristics

**Fig. 1:**
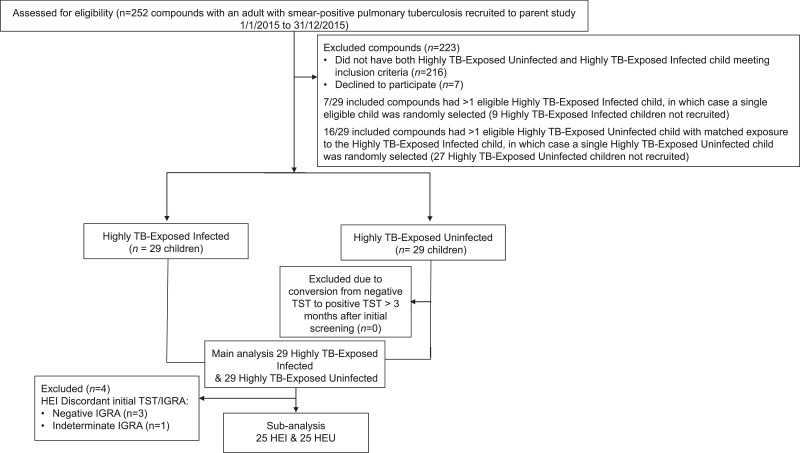
Study recruitment profile. TST = Tuberculin Skin Test, IGRA = Interferon Gamma Release Assay.

A total of 58 children were recruited to the study from 29 different residential compounds. The study recruitment profile is shown in Fig. 1. Demographic features of the study participants together with descriptive statistics are detailed in Table 1. Highly TB-Exposed Infected children were significantly older than Highly TB-Exposed Uninfected children (Highly TB-Exposed Infected: 10.37, interquartile range (IQR) 9.43–12.36; Highly TB-Exposed Uninfected:7.89, IQR: 7.01–10.86, *p* = 0.027). 54 (93%) of the children were sleeping in the same house as the adult index case, and 4 (7%) were sleeping in the same room as the adult index case. The proportion of study participants where the adult index case was their parent did not significantly differ between groups (Highly TB-Exposed Infected children: 7/29, Highly TB-Exposed Uninfected children: 4/29; *p* = 0.45). Highly TB-Exposed Infected children were significantly more likely to be a sibling of the adult (>15 years old) index case (10/29) than Highly TB-Exposed Uninfected children (3/29, *p* = 0.02). Highly TB-Exposed Infected children had a median TST of 18 mm with a range of 13 mm to 25 mm (interquartile range 16 to 20 mm). All 29 Highly TB-Exposed Uninfected children had a TST of 0 mm both at baseline and between 3 and 12 months later. None of the Highly TB-Exposed Infected children who were seen in clinic had HIV infection and 28/29 of the adult index cases had negative HIV tests. The index case who did not consent for HIV testing was not a parent of the Highly TB-Exposed Infected or Highly TB-Exposed Uninfected children. 25 of the 29 Highly TB-Exposed Infected children had a positive in-house IGRA at the initial clinic visit concordant with their positive TST result. 3 Highly TB-Exposed Infected children had a negative IGRA and 1 had an indeterminate IGRA at their initial clinic visit (Fig. 1). There were no significant differences in haematological parameters, including monocyte: lymphocyte ratio, between Highly TB-Exposed Infected and Highly TB-Exposed Uninfected groups (Table 1). No participant developed tuberculosis during 12 months of follow-up.

**Table 1:** Summary of participant characteristics.

Characteristic		Highly TB-exposed uninfected children	Highly TB-exposed infected children	*p*
*n*		29	29	–
Sleeping proximity	Same room	2	2	–
	Same house	27	27	–
	Different house	0	0	–
Median age in years (IQR)		7.89 (7.01–10.86)	10.37 (9.43–12.36)	**0.027**^a^
% Males		14 (48)	16 (55)	0.62^b^
HIV infection in child (*n* = 29)		–	0	–
HIV infection in adult index case (adult consented to test *n* = 28)		0		
Relationship of child to adult index case (%)	Child	4 (13.8)	7 (24.1)	0.45^b^
	Cousin	6 (20.7)	2 (6.9)	0.13^b^
	Grandchild	1 (3.4)	2 (6.9)	1.00^b^
	Nephew or Niece	9 (31.0)	6 (20.7)	0.45^b^
	Sibling	3 (10.3)	10 (34.5)	**0.02**^b^
	Distant relation	1 (3.4)	1 (3.4)	1.00^b^
	Unrelated	5 (17.2)	1 (3.4)	0.13^b^
Median initial TST result in mm (IQR)		0 (0)	18 (16–20)	**0.000**^a^
Median repeat TST result in mm (IQR)		0 (0)	–	–
Initial Interferon Gamma Release Assay	Positive	–	25 (86.2%)	–
	Negative	–	3 (10.3%)	–
	Indeterminate	–	1 (0.3%)	–
Median absolute White blood cell count k/µL (IQR)		6.38 (5.58–7.18)	6.02 (5.6–6.75)	0.563^a^
Mean absolute monocyte count k/µL (95% CI)		0.467 (0.381–0.552)	0.443 (0.352–0.534)	0.657^c^
Median absolute neutrophil count k/µL (IQR)		2.44 (1.88–2.83)	2.61 (2.17–3.19)	0.492^a^
Mean absolute lymphocyte count k/µL (95% CI)		2.90 (0.99–4.80)	2.67 (0.91–4.44)	0.391^c^
Mean Monocyte:Lymphocyte ratio (95% CI)		0.174 (0.141–0.206)	0.171 (0.138–0.204)	0.905^a^

a denotes Wilcoxon matched pairs signed rank test.

b McNemar's χ^2^ test.

c denotes paired *t*-test. Haematological statistics based upon results available from CellDyn machine (Highly TB-Exposed Infected *n*=25, Highly TB-Exposed Uninfected *n*=25).

**Fig. 2:**
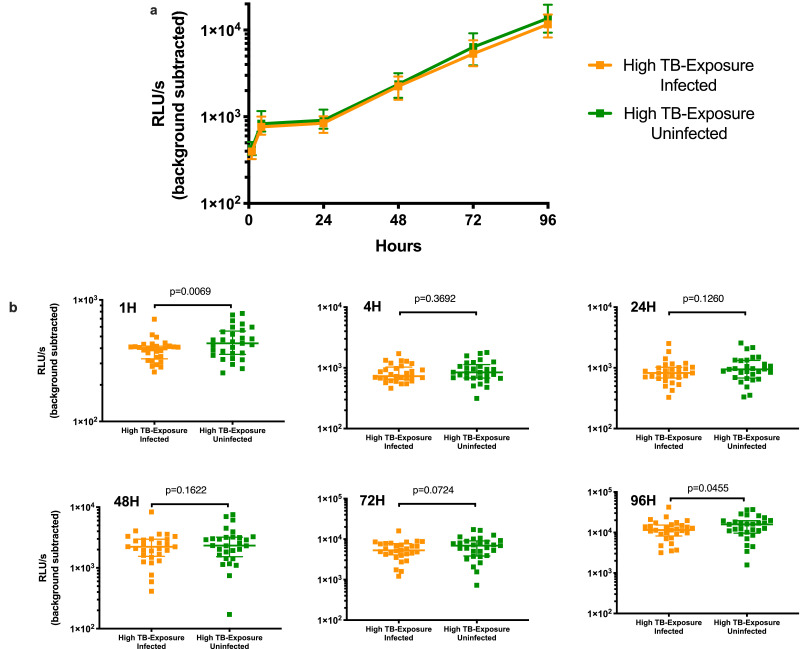
Luminescence kinetics in the whole blood BCG assay for all Highly TB-Exposed Uninfected and Highly TB-Exposed Infected children. a) Graph and b) column scatterplots of the data. Median values and interquartile ranges are shown. *p* values from Wilcoxon matched pairs signed rank test. Scale on y axis for each plot selected to optimise data display.

*In vitro control of BCG bacterial growth:* Bacterial growth in whole blood was measured by luminescence at 6 timepoints, resulting in longitudinal bacterial growth profiles in blood from all 58 participants. Infected blood from Highly TB-Exposed Infected children had 8.5% lower luminescence at baseline (median 402.4, IQR: 327.6 - 416.7 RLU/s) compared to Highly TB-Exposed Uninfected children (median 439.7, IQR: 356.4 - 554 RLU/s; *p* = 0.0069). This difference was not statistically significant at intermediate timepoints, but at 96 hours, there was 38% lower bacterial luminescence in samples from Highly TB-Exposed Infected (median 11,511, IQR: 8210–15,272 RLU/s) compared to Highly TB-Exposed Uninfected children (median 15,895, IQR 9655–19,585 RLU/s; *p* = 0.0455) (Fig. 2, Supplementary Fig. 2). Mixed effects modelling, treating age, whether the child was a sibling of the index case, and other variables as balanced between groups, modelled Highly TB-Exposed Infected children to have lower luminescence than Highly TB-Exposed Uninfected children at baseline, although this was not statistically significant (Highly TB-Exposed Infected: Modelled estimated marginal mean (MEMM): 400.8, 95% CI: 332.7–482.8 RLU/s; Highly TB-Exposed Uninfected: MEMM: 456.6, 95%CI 374.8–556.2 RLU/s; *p* value=0.09, Table 2 and Supplementary Table 3). Highly TB-Exposed Infected children had significantly lower modelled luminescence than Highly TB-Exposed Uninfected children at 96 hours (Highly TB-Exposed Infected: MEMM: 11,262 RLU/s, 95% CI: 9347–13,568; Highly TB-Exposed Uninfected MEMM: 13,304 RLU/s, 95% CI: 10,921–16,206, *p* = 0.031). Similar patterns were seen in the sub-analysis where the Highly TB-Exposed Infected children with negative or indeterminate baseline IGRAs where excluded (Supplementary Fig. 3).

**Table 2:** Modelled estimated marginal means for luminescence of Highly TB-Exposed Infected and Highly TB-Exposed Uninfected pairs of children Estimated marginal means and 95% confidence intervals by group and time were predicted from the model treating all fixed effects including age as balanced. *P* values derived from pairwise comparisons of adjusted predictions.

	Modelled estimated marginal means for luminescence in relative light units/second (95%CI)		
Experimental timepoint	Highly TB-exposed Infected (95% CI)	Highly TB-exposed Uninfected (95% CI)	*p*
Baseline	400.78 (332.66–482.84)	456.59 (374.81–556.21)	0.092
4 h	848.10 (703.95–1,021.76)	889.31 (730.02–1,083.34)	0.54
24 h	872.47 (724.18–1,051.12)	970.13 (796.37–1,181.80)	0.171
48 h	2,144.54 (1,780.05–2,583.66)	2,307.49 (1,894.20–2,810.95)	0.345
72 h	5,282.15 (4,384.38–6,363.76)	6,005.02 (4,929.47–7,315.24)	0.098
96 h	11,261.95 (9,347.84–13,568.01)	13,303.89 (10,921.06–16,206.62)	**0.031**

**Fig. 3:**
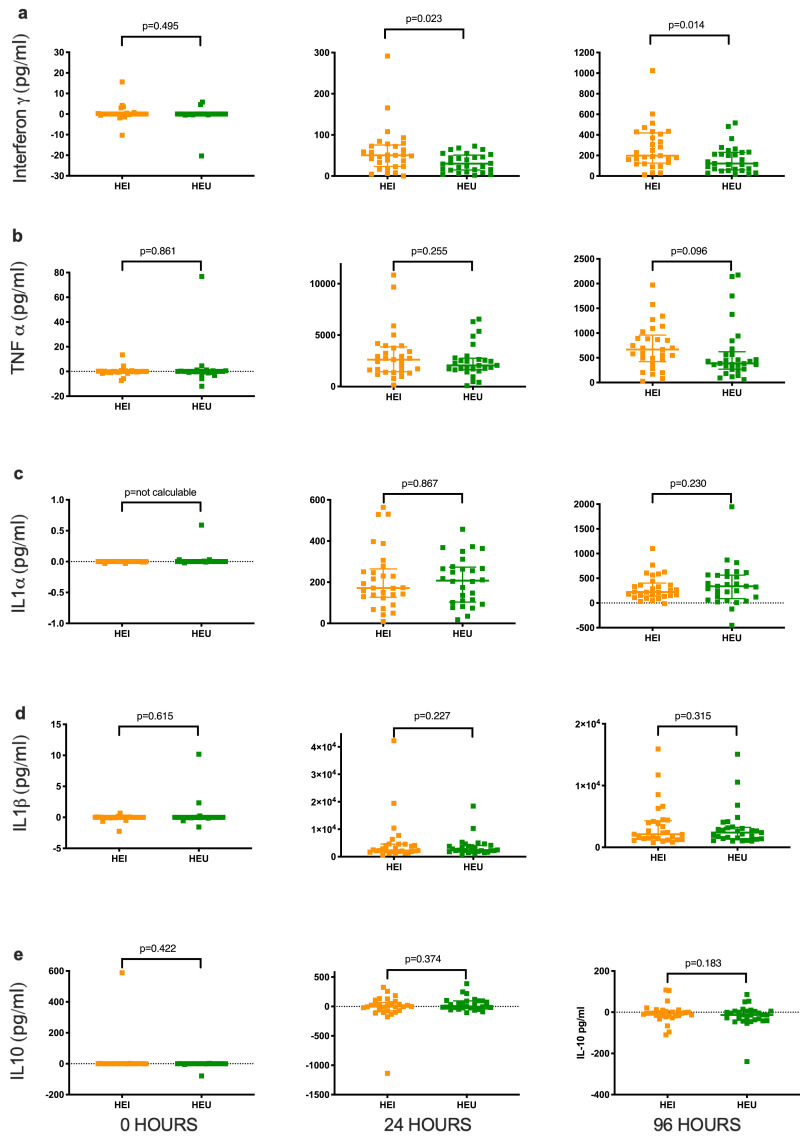
BCG-specific cytokine levels for the 29 Highly TB-Exposed Infected and 29 Highly TB-Exposed Uninfected children. a) IFNγ, b) TNFα, b) IL1α, d) IL1β, and e) IL10. Median and interquartile ranges. *P* values from Wilcoxon matched pairs signed rank test. Scale on y axis for each plot selected to optimise data display.

*Cytokine responses to BCG bacteria in Highly TB-Exposed Uninfected versus Highly TB-Exposed Infected children:* Data were available for all 58 children for baseline and 96-hour timepoints. There was insufficient sample from one Highly TB-Exposed Uninfected child for the 24-h timepoint, and therefore data from 28 pairs was analysed for this timepoint. After the baseline timepoint, all cytokine levels were quantifiable in response to *in vitro* infection with BCG, except for IFN-γ in one 24-hour timepoint sample. BCG-specific IFN-γ responses were higher in blood from Highly TB-Exposed Infected children at 24 h (median 50.44 pg/ml, IQR 23.15–75.36) than Highly TB-Exposed Uninfected children (median 30.06pg/ml, IQR 14.64–50.86, *p* = 0.0232, Fig. 3a, Table 3, Supplementary Fig. 4). Highly TB-Exposed Infected children also had higher levels of BCG-specific IFN-γ at 96 h (median 197.2 pg/ml, IQR 125.4–418.3) compared to Highly TB-Exposed Uninfected children (median 121.4pg/ml, IQR 59.53–231; *p* = 0.0138). The BCG-specific IFN-γ responses were similar between the 25 Highly TB-Exposed Infected children with a concordant positive IGRA and those 4 Highly TB-Exposed Infected children with discordant negative or indeterminate IGRA (Supplementary Fig. 5). TNFα responses were not significantly different in Highly TB-Exposed Infected children at 24 h (median 2605 pg/ml, IQR 1435–3850) compared to Highly TB-Exposed Infected children (median 2053 pg/ml, IQR 1635–2746, *p* = 0.2545, Fig. 3b, Table 3)). At 96 h, TNFα responses were higher in Highly TB-Exposed Infected children (median 667.6pg/ml, IQR 421.4–959.2) than Highly TB-Exposed Uninfected children (median: 386pg/ml, IQR 268.8–622.3) although this was not statistically significant (*p* = 0.096) There were no statistically significant differences between IL1α, IL1β, and IL10 responses to BCG in whole blood from Highly TB-Exposed Uninfected and Highly TB-Exposed Infected children (Fig. 3c–e, Table 3).

**Table 3:** BCG-specific cytokine levels for the Highly TB-Exposed Infected and Highly TB-Exposed Uninfected pairs of children.

	0 h			24 h^a^			96 h		
	Highly TB-exposed infected	Highly TB-exposed uninfected	*p*	Highly TB-exposed infected	Highly TB-exposed uninfected	*p*	Highly TB-exposed infected	Highly TB-exposed uninfected	*p*
IFNγ (pg/ml)	0 (0–0.135)	0 (0–0)	0.495	50.44 (23.2–75.4)	30.06 (14.6–50.9)	0.023	197.2 (125.4–418.3)	121.4 (59.5–231)	0.014
TNFα (pg/ml)	0 (−0.55–0.25)	0 (−0.52–0.15)	0.861	2605 (1435–3850)	2053 (1635–2746)	0.254	667.6 (421.4–959.2)	386 (268.8–622.3)	0.096
IL1α (pg/ml)	0 (0–0)	0 (0–0)	-b	171.6 (126.9–264.6)	207.7 (103.7–273.5)	0.867	221.1 (143.3–403.1)	337.7 (88.8–562.8)	0.230
IL1β (pg/ml)	0 (−0.03–0)	0 (−0.03–0)	0.615	2246 (1849–4584)	2419 (1863–4064)	0.227	2120 (1342–4313)	2400 (1405–3236)	0.315
IL10 (pg/ml)	0 (−0.20–0.24)	0 (−0.30–0.07)	0.422	−1.61 (−73.6–68.2)	−3.74 (−25.8–94.4)	0.374	−5.43 (−18.7–7.45)	−13.05 (−38.8–5.09)	0.183

a for this timepoint data available for 28 complete pairs.

b 21 of 29 pairs had no detectable BCG-specific IL1α at baseline and therefore an accurate *p* value for the Wilcoxon matched pairs signed rank test could not be calculated.

## 4 Discussion

Given the importance of understanding human protective immunity to LTBI to guide vaccine design and evaluation, we applied a novel functional mycobacterial assay in a tuberculosis-endemic country to children with a persistently negative TST despite defined household MTB exposure. We conducted a carefully matched study of children with discordant infection status despite the same sleeping proximity to the same adult with smear-positive pulmonary tuberculosis. We utilised a whole blood autoluminescent BCG growth assay to identify differences in functional control of mycobacteria and cytokine responses in these individuals that could correlate with protection against infection. The potential to identify functional differences in the dynamics of host-mycobacterial interactions using small volumes of blood is a major advantage of the autoluminescent properties harnessed in this method, and is not possible with other whole blood mycobacterial assays. [25,26,29,39,47,48]

We hypothesised that Highly TB-Exposed Uninfected children would exhibit superior mycobacterial control as measured by lower experimental luminescence at early experimental timepoints than Highly TB-Exposed Infected children. Non-parametric bivariate tests of luminescence one hour after infection instead showed a statistically significant opposite effect. The observation was not significant in mixed effects modelling incorporating the longitudinal nature of the data, age, and inter-individual variability, so further investigation is needed. The possible differences within only one hour after infection of samples suggest further research focusing on the trained innate immune response may be of value [18,49,50]. A difference in inoculum is unlikely to account for the differences in baseline and 96 h luminescence between the groups as all samples from a pair were handled in the same way and inoculated with the same volume from the same stock of BCG at the same time. Baseline luminescence was measured one hour after inoculation to allow samples to equilibrate to 37 °C in the incubator, and each triplicate tube was individually removed, measured, and returned, so there was a maximum ten-minute interval between measuring samples from the two children in a pair [39]. We did not see differences in cytokine response at baseline. Both non-parametric statistics and mixed effects modelling showed significantly greater mycobacterial control by Highly TB-Exposed Infected children at 96 h, mirroring results from two adult studies, which used different functional mycobacterial growth assays and suggest that MGIA are likely measuring adaptive responses at this time-point [25,29,51]. However, a comparison of unmatched groups of 20 IGRA-positive and 28 IGRA-negative 8 year-old South African children did not demonstrate a difference in mycobacterial growth inhibition [48]. Our luminescence data did not support superior mycobacterial control by Highly TB-Exposed Uninfected children and we therefore rejected our hypothesis and found that luminescence was not a correlate of protection in the assay's current form.

The TST phenotype was dramatically different between the Highly TB-Exposed Infected and Highly TB-Exposed Uninfected groups, with Highly TB-Exposed Infected children strongly positive, and all Highly TB-Exposed Uninfected children consistently having 0 mm results, tested twice with a minimum of three months intervals. We found that Highly TB-Exposed Infected children were older than Highly TB-Exposed Uninfected children, in keeping with data from prevalence studies, although the absolute difference in median age of 2.5 years in a study where all participants are older than five is unlikely to explain such dramatic differences in TST results [5,33,52]. Highly TB-Exposed Infected children were also significantly more likely to be a sibling of the adult index case, which may have implications for the nature of interactions with, and hence exposure to, the index case. Both age and sibling status were included as variables in the mixed effects modelling of luminescence data.

To our knowledge, our study is the first to apply a matched study design to the evaluation of whole blood cytokine responses to mycobacteria in children without LTBI despite exposure. Highly TB-Exposed Infected children produced significantly more IFN-γ in response to BCG at 24 and 96 hours. IFN-γ is recognised as a necessary but not sufficient mediator of host response to mycobacteria and is of course the basis of IGRAs [5]. In keeping with our findings, in two distinct cohorts in Uganda and Pakistan, IFN-γ levels in response to mycobacterial stimulation were also found to be lower in persistently TST negative individuals compared to those with LTBI or those who convert to a positive TST [53], [54], [55].

We did not find statistically significant differences in TNF-α levels as has also been previously observed [53,54,56]. The absence of significantly different cytokine results between groups are also in concordance with other published studies. Although there was not a matched design and the number of paediatric participants is not clear, persistently IGRA negative Indonesian household TB contacts in comparison to those who converted to a positive IGRA showed no difference in levels of TNF-α, IL1β, IL-1RA, IL-10, IL-6 and IL-8 in response to *in vitro* BCG infection or stimulation with an MTB lysate [18]. There was also no difference in IL-2, IFN-γ, TNF-α, and/or IL- 17-expressing subsets of CD4, CD8 and γδ T-cells between IGRA+ and IGRA- South African adults following 12 h of BCG stimulation, nor correlations with MTB growth in a cross-sectional study [48]. Cytokine responses to MTB antigens of samples from babies born to mothers with or without LTBI also do not differ [57].

Our study had notable strengths in its matched design, clear differences in the TST phenotype, and confirmation of persistent TST negativity for at least 3 months in the Highly TB-Exposed Uninfected children. The application of the novel autoluminescent BCG whole blood assay enabled the possibility to examine the kinetics of host-mycobacterial interactions in children using minimal blood volumes. However, our study also had limitations. The sample size was not large, although the number of children was greater or comparable to other studies in the field [18,48]. Due to the limited sample capacity of laboratory equipment and the importance of simultaneous conduct of the functional mycobacterial assay on samples from matched children in the same experiment, it was not possible to recruit every Highly TB-Exposed Infected and Uninfected child. The study was conducted in a single centre. IGRA status was only available at baseline for the Highly TB-Exposed Infected children and was not available for the Highly TB-Exposed Uninfected children. Nevertheless, excluding the four IGRA/TST discordant Highly TB-Exposed Infected children did not affect the results. Also, all of the Highly TB-Exposed Uninfected children maintained their TST results of 0 mm at baseline and at least 3 months later and the likelihood of a positive IGRA would be low. The Highly TB-Exposed Infected children were classified as such following initial screening; therefore, we cannot be certain whether their infection status was secondary to the identified recent household exposure or more longstanding. Data on additional epidemiological factors such as ventilation, use of mosquito nets, school attendance and movement within and outside the household are not available. With regard to the immune status of the children, no HIV infection was identified in the Highly TB-Exposed Infected children or the adult index cases who were tested. Although the study design meant that within matched pairs of children exposure was to the same index case (and hence strain of MTB), mycobacterial culture on adults with pulmonary tuberculosis was not routine within the Gambian National Leprosy and Tuberculosis Control Program so data on MTB lineage and strains in the index cases are not available. Our study used BCG as the mycobacterial stimulus rather than pathogenic MTB. However, inoculum strain has been shown not to be a major determinant in mycobacterial growth inhibition assays in a high TB-prevalence setting, where BCG, MTB H37Rv, a W/Beijing MTB strain HN878, and Euro-American MTB strain, CDC1551 were all used to compare individuals with and without LTBI, including children and young adults [48]. The experimental stimulus being BCG in a population with near universal coverage of neonatal BCG vaccine, may have contributed to the similarity of cytokine responses between groups, except for IFN-γ levels that matched infection status. Nevertheless, the significant difference in luminescence at 96 h between groups suggests that even against this background of BCG vaccination and environmental mycobacterial exposure, the assay is able to distinguish children by their LTBI status. It may be that we were not able to detect differences in luminescence at early timepoints due to the need for a relatively high multiplicity of infection, as a consequence of the relatively low autoluminescence of the bacteria, in order for baseline measurements to be within the dynamic range of the luminometer [39].

We have demonstrated the importance of careful exposure-matched study design when comparing experimental data on samples from patients with LTBI to those who remain uninfected despite TB contact. We would encourage such study designs to be adopted more widely. We have also employed for the first time an autoluminescent mycobacterial growth assay specifically designed for paediatric studies where serial non-destructive luminescence measurements and supernatant analysis were possible with less than 5mls total blood. In conclusion, we found no evidence for a superior ability to restrict mycobacterial growth *in vitro* in children who were MTB exposed but remained uninfected, compared to their well-matched counterparts. We also did not identify any key pro-inflammatory cytokine responses to BCG associated with remaining uninfected. Identifying correlates of protection against LTBI remains challenging. We suggest a continued research focus on persistently TST negative individuals despite exposure, with application of novel and improved functional assays, broader investigation of trained innate immunity, antibody effector function, gene expression and genetic diversity, and metabolomic differences needed to yield further biological insights that can be applied to guide vaccine design and evaluation [3,4,18,20,50,58].

## Declaration of Competing Interest

BK reports grants from MRC UK, during the conduct of the study; In addition, BK has a patent for a paediatric biomarker signature issued (United Kingdom Patent Application Number 1602305). The authors do not have any other commercial or other association that might pose a conflict of interest. The data presented here form part of RB's PhD thesis “Protection from *Mycobacterium tuberculosis* infection: Learning from exposed but uninfected children” awarded March 2018 by Imperial College London.

## Appendix Supplementary materials

Image, application 1
