# Supplementary material for: Protection against mycobacterial infection: A case-control study of mycobacterial immune responses in pairs of Gambian children with discordant infection status despite matched TB exposure
Source: eBioMedicine. 2020 Jul 13;59:102891. doi: 10.1016/j.ebiom.2020.102891 (PMC7502674; doi:10.1016/j.ebiom.2020.102891)
Supplement: Supplementary file 1 [file mmc1.pdf]

SUPPLEMENTARY MATERIALS

Supplementary Table 1: Case definitions for children recruited to the study

Supplementary Table 2: Inclusion and exclusion criteria for identification of household compounds to participate in this study.

Supplementary Table 3: Regression coefficients (β) of fixed effects and estimates for random effects for predicting log luminescence according to a linear mixed model. The models were run with the unstructured covariance option and were fitted via restricted maximum likelihood. [40] The distribution of residuals was checked by histograms and quantile-normal plots.

Supplementary Figure 1: Inhibition of growth of BCG LuxAB by TB-Exposed Uninfected children compared to TB-Exposed Infected children from a pilot household contact study in Cape Town, South Africa.
Whole blood was incubated with BCG-luxAB and metabolically active bacteria were measured at time 0 and 96 hours by bioluminescence. A growth ratio was calculated (T96/T0). TB-exposed infected children (as defined by positive TST, n=18) and TB-exposed but uninfected children (as defined by negative TST, n=24) were compared. Matched statistical analysis was not possible. Age did not significantly differ between the groups (Children with infection: n=14, median: 10.5 years, IQR:3.75-13.25; Children without infection: n=23, median: 6 years, IQR: 4-10; p=0.252).Horizontal lines=median, error bars=interquartile range, p values from two-tailed Mann-Whitney U test (Previously unpublished data B Kampmann)
Supplementary Figure 2: Column scatterplots of the luminescence kinetics in the whole blood BCG assay for all Highly TB-Exposed Uninfected and Highly TB-

Exposed Infected children from Figure 2b with datapoints from each pair connected by a coloured line. Median values and interquartile ranges are shown. *p* values from Wilcoxon matched pairs signed rank test. Scale on y axis for each plot selected to optimize data display.

Supplementary Figure 3: Luminescence kinetics in the whole blood BCG assay showing only pairs of Highly TB-Exposed Uninfected and Highly TB-Exposed Infected children where Highly TB-Exposed Infected children had a positive in-house IGRA result.
A) Graph and B) column scatterplots of the data. Median values and interquartile ranges are shown. *p* values from Wilcoxon matched pairs signed rank test.

Supplementary Figure 4: Column scatterplots of the BCG-specific cytokine levels for the 29 Highly TB-Exposed Infected and 29 Highly TB-Exposed Uninfected children from Figure 3 with datapoints from each pair connected by a coloured line.
a) IFNγ, b) TNFα, b) IL1α, d) IL1β, and e) IL10. Median and interquartile ranges. P values from Wilcoxon matched pairs signed rank test. Scale on y axis for each plot selected to optimize data display.

Supplementary Figure 5: BCG-specific Interferon Gamma levels by IGRA result for the 29 Highly TB-Exposed Infected children, comparing 25 IGRA positive Highly TB-Exposed Infected children to 4 Highly TB-Exposed Infected children with negative or indeterminate IGRAs.

STROBE Checklist

Supplementary Table 1: Case definitions for children recruited to the study

**Child classification**	**High Exposure Infected**	**High Exposure Uninfected**
**Age**	5 years ≤ Age < 15 years	5 years ≤ Age < 15 years
**Exposure to adult index case with smear-positive tuberculosis**	Sleeps in same bed, same room, or same house	Same sleeping proximity as Highly TB-Exposed Infected child.
**Infection status**	Initial TST ≥10mm	Initial TST ≤5mm *and* repeat TST >3 months later ≤5mm
**Disease status**	Asymptomatic on symptom screening and clinical evaluation including normal chest radiograph	Asymptomatic on symptom screening

Supplementary Table 2: Inclusion and exclusion criteria for identification of household compounds to participate in this study.

Inclusion Criteria	Exclusion Criteria
More than two child contacts (age 5 years→15 years) of the index case	Children being considered for this study have previously received treatment for TB infection or disease
Index case has smear positive tuberculosis	
Family lives in the Greater Banjul Area	Family is planning to leave Greater Banjul Area in next 12 months
The child contacts have had Tuberculin Skin Test (TST) placed, read, and recorded	
There is at least one child contact with TST greater than or equal to 10mm	All the children with TST greater than or equal to 10mm have symptoms of TB
There is at least one child with TST less than or equal to 5mm	All the children with TST less than or equal to 5mm have symptoms of TB
There is a parent or guardian who can give consent	

Supplementary Table 3: Regression coefficients (β) of fixed effects and estimates for random effects for predicting log luminescence according to a linear mixed model

Fixed Effects:					
		Coefficient	Standard Error	p	95% CI
Highly TB-Exposed Uninfected compared to Highly TB-Exposed Infected		0.13	0.077	0.09	-0.02-0.28
Sibling of index case (binary variable)		0.17	0.12	0.16	-0.07-0.41
Age (compared to youngest tertile)	Middle tertile	-0.17	0.10	0.86	-0.20-0.17
	Oldest tertile	-0.20	0.11	0.06	-0.40-0.01
Experimental timepoint (compared to baseline)	4H	0.75	0.049	0	0.65-0.85
	24H	0.78	0.049	0	0.68-0.87
	48H	1.68	0.049	0	1.58-1.77
	72H	2.58	0.049	0	2.48-2.68
	96H	3.34	0.049	0	3.23-3.43
Interaction between infection status (Highly TB-Exposed Uninfected compared to Highly TB-Exposed Infected) and experimental timepoint (compared to baseline)	4H	-0.083	0.07	0.23	-0.22-0.053
	24H	-0.024	0.07	0.73	-0.16-0.11
	48H	-0.057	0.07	0.41	-0.19-0.079
	72H	-0.0021	0.07	0.98	-0.14-0.13
	96H	0.036	0.07	0.60	-0.10-0.17
Constant		5.98	0.11	0	5.75-6.20

Random Effects			
	Estimate	Standard Error	95% CI
Pair of children	0.41	0.064	0.30-0.55
Individual child	0.19	0.031	0.14-0.26
Residual	0.32	0.073	0.31-0.34

Supplementary Figure 1: Inhibition of growth of BCG LuxAB by TB-Exposed Uninfected children compared to TB-Exposed Infected children from a pilot household contact study in Cape Town, South Africa.

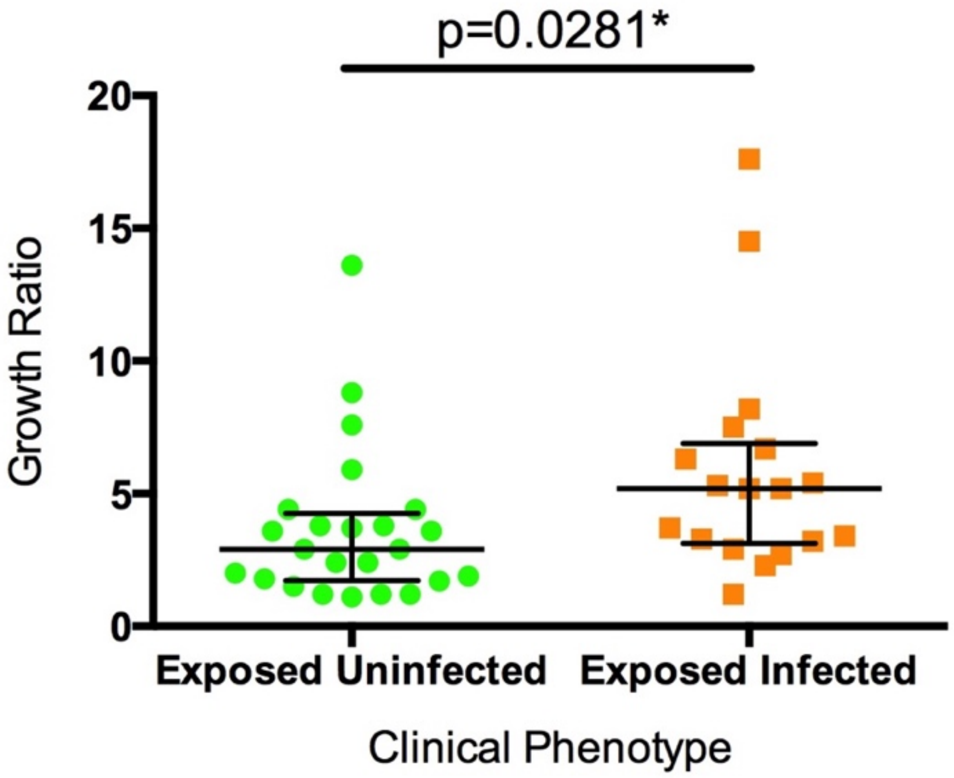

Supplementary Figure 2: Column scatterplots with datapoints from each pair connected by a coloured line of the luminescence kinetics in the whole blood BCG assay for all Highly TB-Exposed Uninfected and Highly TB-Exposed Infected children from Figure 2b.

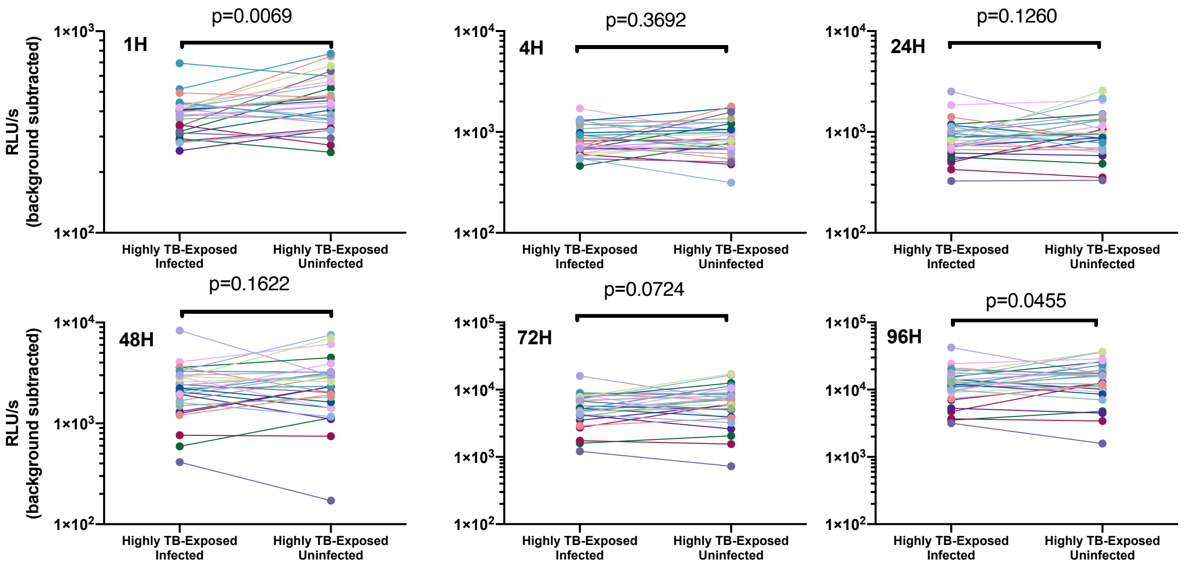

Supplementary Figure 3: Luminescence kinetics in the whole blood BCG assay showing only data from 25 pairs of Highly TB-Exposed Uninfected and Highly TB-Exposed Infected children where Highly TB-Exposed Infected children had a positive in-house IGRA result.

a:

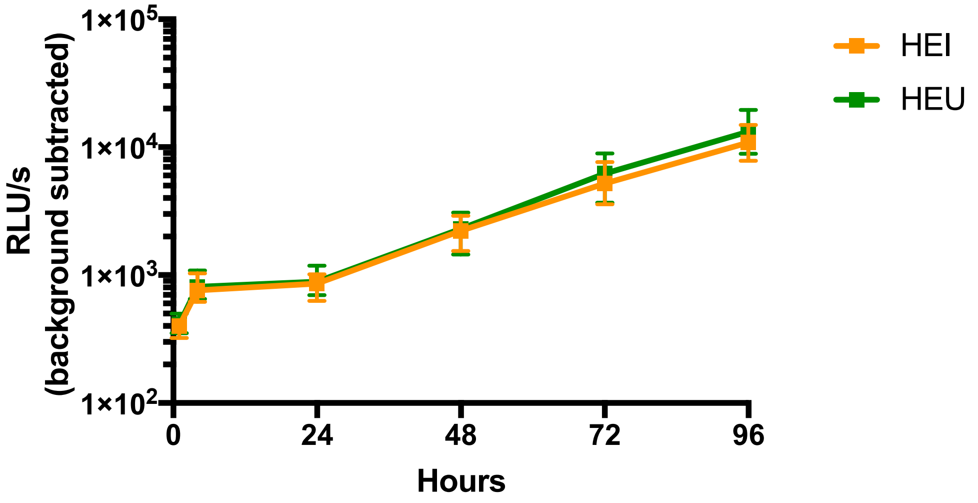

b:

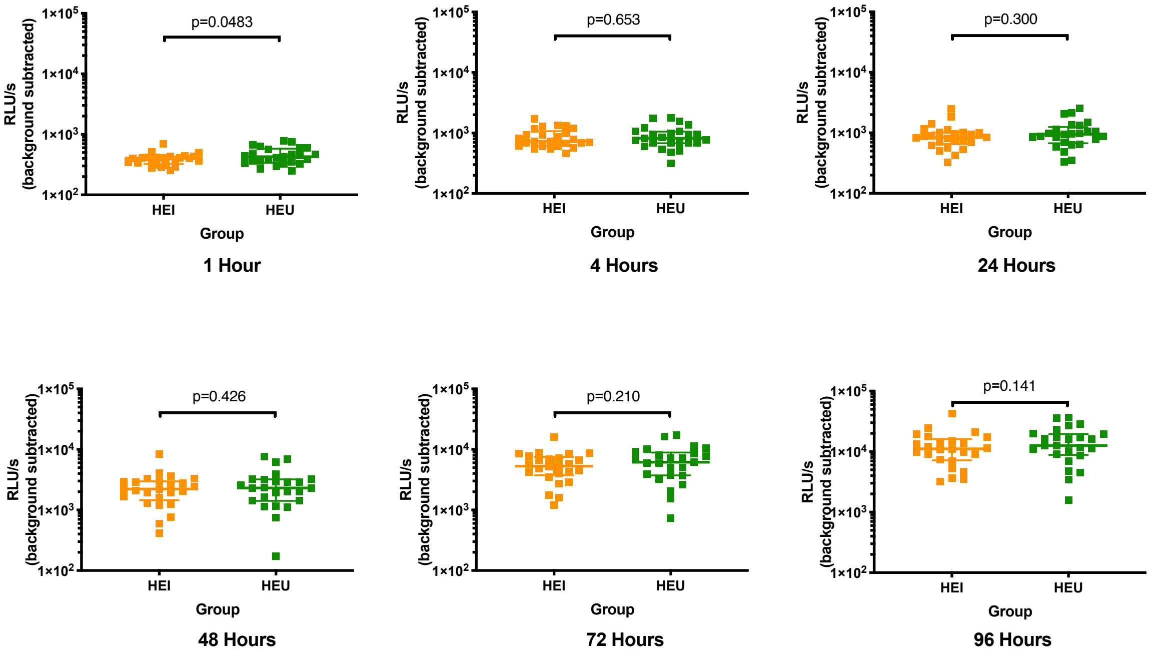

Supplementary Figure 4: Column scatterplots with datapoints from each pair connected by a coloured line of the BCG-specific cytokine levels for the 29 Highly TB-Exposed Infected and 29 Highly TB-Exposed Uninfected children from Figure 3. a) IFNγ, b) TNFα, b) IL1α, d) IL1β, and e) IL10.

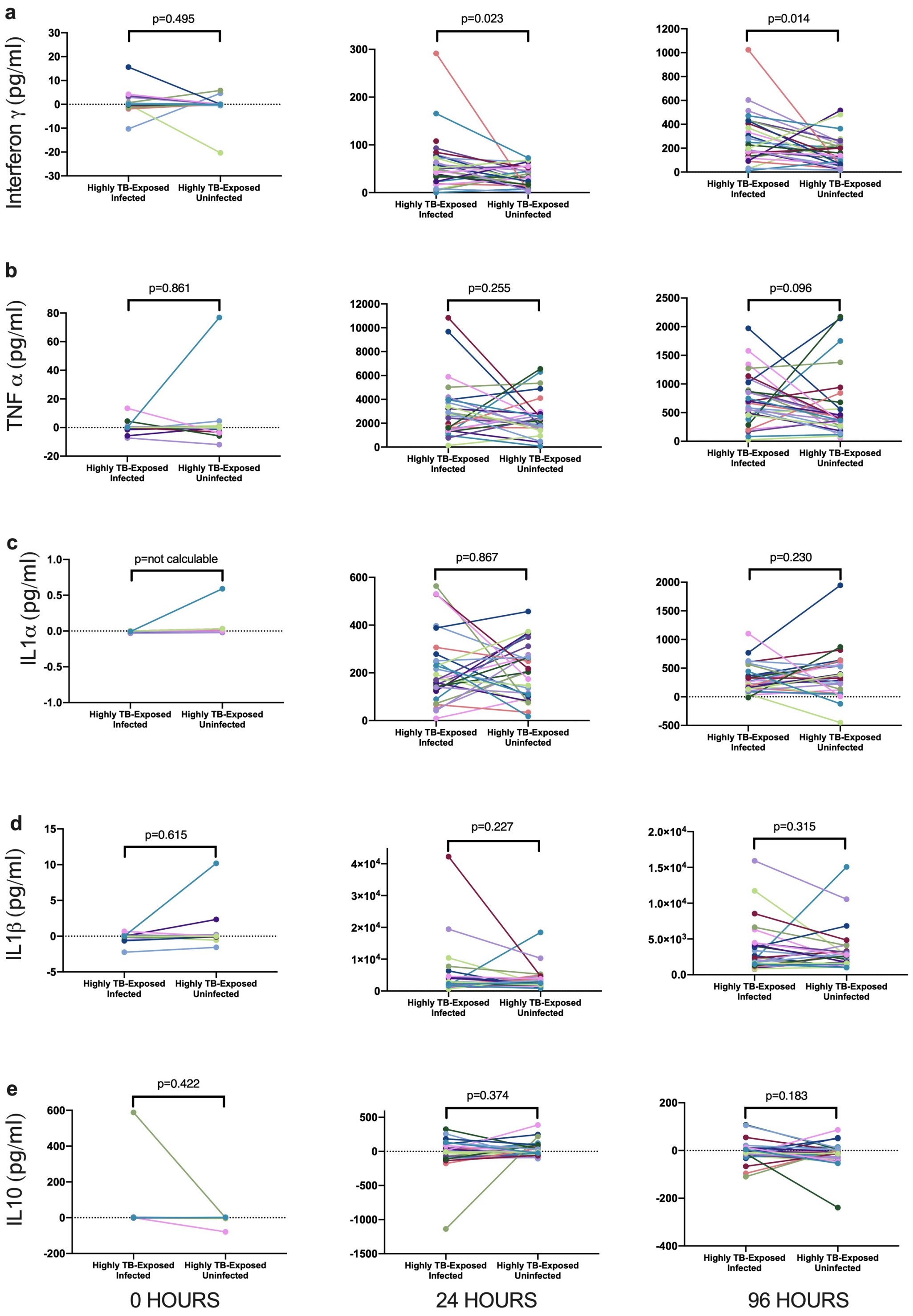

Supplementary Figure 5: BCG-specific Interferon Gamma levels by IGRA result for the 29 HEI children, comparing 25 IGRA positive Highly TB-Exposed Infected children to 4 Highly TB-Exposed Infected children with negative or indeterminate IGRAs.

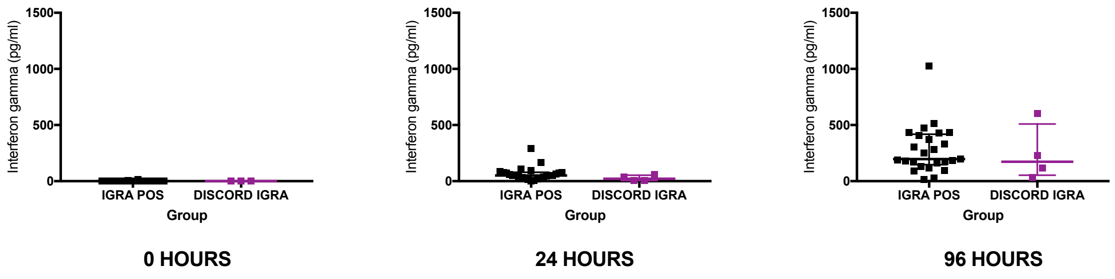

STROBE Statement—Checklist of items that should be included in reports of ***case-control studies*** (page numbers apply to original manuscript prior to proof/PDF version)

	Item No	Recommendation	Page
**Title and abstract**	1	(*a*) Indicate the study's design with a commonly used term in the title or the abstract	1-2
		(*b*) Provide in the abstract an informative and balanced summary of what was done and what was found	2
**Introduction**			
Background/rationale	2	Explain the scientific background and rationale for the investigation being reported	4-6
Objectives	3	State specific objectives, including any prespecified hypotheses	6
**Methods**			
Study design	4	Present key elements of study design early in the paper	7-11
Setting	5	Describe the setting, locations, and relevant dates, including periods of recruitment, exposure, follow-up, and data collection	7-8
Participants	6	(*a*) Give the eligibility criteria, and the sources and methods of case ascertainment and control selection. Give the rationale for the choice of cases and controls	7-8 Sup Tab 1+2
		(*b*) For matched studies, give matching criteria and the number of controls per case	7-8
Variables	7	Clearly define all outcomes, exposures, predictors, potential confounders, and effect modifiers. Give diagnostic criteria, if applicable	11 Sup Tab 1+2
Data sources/ measurement	8*	For each variable of interest, give sources of data and details of methods of assessment (measurement). Describe comparability of assessment methods if there is more than one group	7-9 Sup Tab 1+2
Bias	9	Describe any efforts to address potential sources of bias	5-6, 11
Study size	10	Explain how the study size was arrived at	7
Quantitative variables	11	Explain how quantitative variables were handled in the analyses. If applicable, describe which groupings were chosen and why	11
Statistical methods	12	(*a*) Describe all statistical methods, including those used to control for confounding	11
		(*b*) Describe any methods used to examine subgroups and interactions	11
		(*c*) Explain how missing data were addressed	12, 14
		(*d*) If applicable, explain how matching of cases and controls was addressed	11
		(*e*) Describe any sensitivity analyses	N/A
**Results**			
Participants	13*	(a) Report numbers of individuals at each stage of study—eg numbers potentially eligible, examined for eligibility, confirmed eligible, included in the study, completing follow-up, and analysed	Fig 1
		(b) Give reasons for non-participation at each stage	Fig 1
		(c) Consider use of a flow diagram	Fig 1

Descriptive data	14*	(a) Give characteristics of study participants (eg demographic, clinical, social) and information on exposures and potential confounders	12, Table 1
		(b) Indicate number of participants with missing data for each variable of interest	12,13
Outcome data	15*	Report numbers in each exposure category, or summary measures of exposure	12, Table 1
Main results	16	(*a*) Give unadjusted estimates and, if applicable, confounder-adjusted estimates and their precision (eg, 95% confidence interval). Make clear which confounders were adjusted for and why they were included	12-13, Fig 2, Table 2
		(*b*) Report category boundaries when continuous variables were categorized	11
		(*c*) If relevant, consider translating estimates of relative risk into absolute risk for a meaningful time period	N/A
Other analyses	17	Report other analyses done—eg analyses of subgroups and interactions, and sensitivity analyses	Sup Fig 3, 5
**Discussion**			
Key results	18	Summarise key results with reference to study objectives	15-18
Limitations	19	Discuss limitations of the study, taking into account sources of potential bias or imprecision. Discuss both direction and magnitude of any potential bias	18-20
Interpretation	20	Give a cautious overall interpretation of results considering objectives, limitations, multiplicity of analyses, results from similar studies, and other relevant evidence	15-20
Generalisability	21	Discuss the generalisability (external validity) of the study results	18
**Other information**			
Funding	22	Give the source of funding and the role of the funders for the present study and, if applicable, for the original study on which the present article is based	2-3, 21

*Give information separately for cases and controls.

Note: An Explanation and Elaboration article discusses each checklist item and gives methodological background and published examples of transparent reporting. The STROBE checklist is best used in conjunction with this article (freely available on the Web sites of PLoS Medicine at http://www.plosmedicine.org/, Annals of Internal Medicine at http://www.annals.org/, and Epidemiology at http://www.epidem.com/). Information on the STROBE Initiative is available at http://www.strobe-statement.org.
